# Supplementary material for: The role of Mediator and Little Elongation Complex in transcription termination
Source: Nat Commun. 2020 Feb 26;11:1063. doi: 10.1038/s41467-020-14849-1 (PMC7044329; doi:10.1038/s41467-020-14849-1)
Supplement: Supplementary file 2 — Supplementary Information [file 41467_2020_14849_MOESM2_ESM.pdf]

Inventory of Supplementary Information of Takahashi et al.

The role of Mediator and Little elongation complex in transcription termination

Hidehisa Takahashi, Amol Ranjan, Shiyuan Chen, Hidefumi Suzuki, Mio Shibata, Tomonori Hirose, Hiroko Hirose, Kazunori Sasaki, Ryota Abe, Kai Chen, Yanfeng He, Ying Zhang, Ichigaku Takigawa, Tadasuke Tsukiyama, Masashi Watanabe, Satoshi Fujii, Midori Iida, Junichi Yamamoto, Yuki Yamaguchi, Yutaka Suzuki, Masaki Matsumoto, Keiichi I. Nakayama, Michael P. Washburn, Anita Saraf, Laurence Florens, Shigeo Sato, Chieri Tomomori-Sato, Ronald C. Conaway, Joan W. Conaway, Shigetsugu Hatakeyama

Supplementary Figures

Supplementary Figure 1 is related to Figure 1.
Supplementary Figure 2 is related to Figure 2.
Supplementary Figure 3 is related to Figure 3.
Supplementary Figure 4 is related to Figure 4.
Supplementary Figure 5 is related to Figure 4.
Supplementary Figure 6 is related to Figure 4.
Supplementary Figure 7 is related to Figure 5.
Supplementary Figure 8 is related to Figure 9.
Supplementary Figure 9 is related to Figure 10.

Supplementary Methods

Oligonucleotides information of primers used for qPCR analysis and CRISPR vector construction

Supplementary References

Supplementary Figures

Supplementary Figure 1

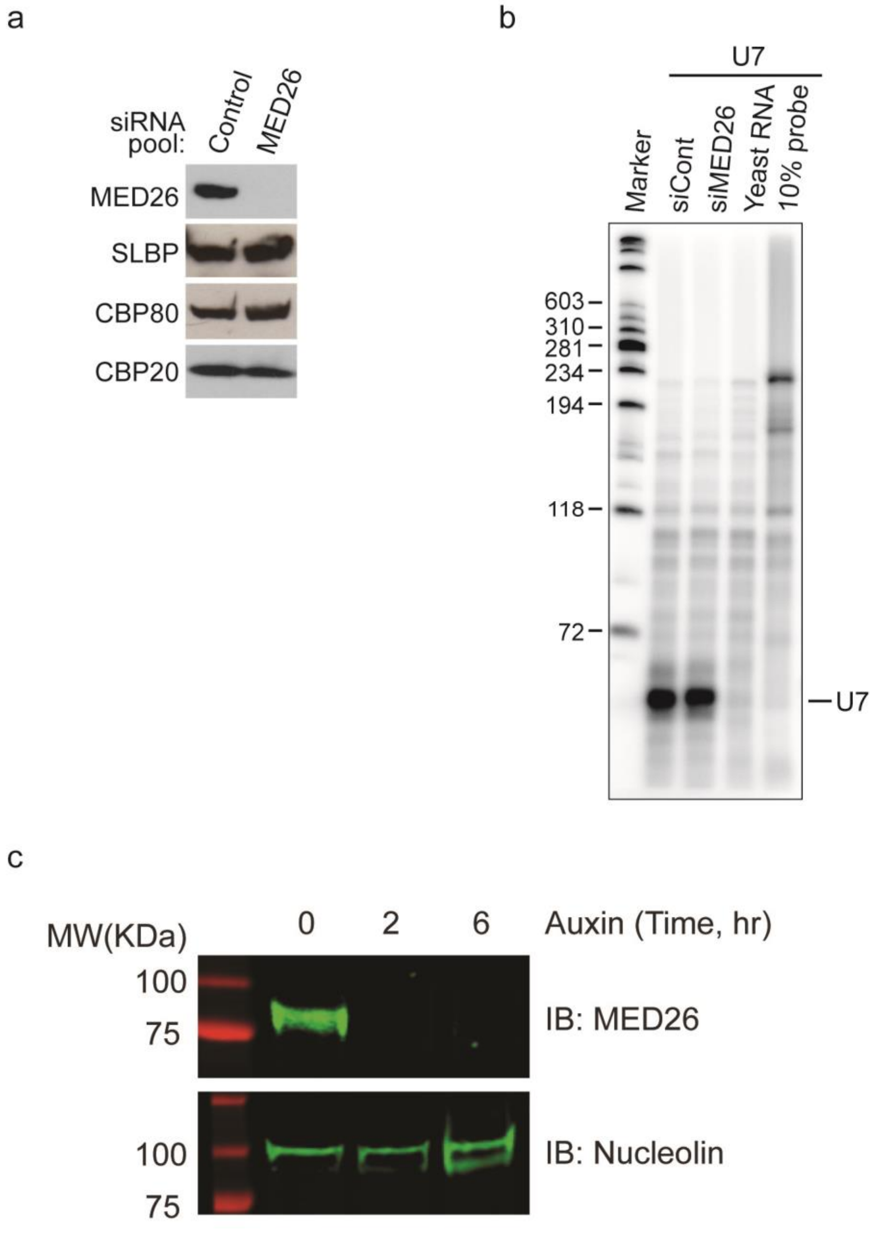

Supplementary Figure 1. MED26 knockdown results in little change in the levels of transcription termination factors. (a) Western blotting showing MED26, SLBP, CBP80, and CBP20 expression 48 h after transfection of nontargeting siRNA or siRNAs targeting MED26. (b) RNase protection assay of U7 snRNA. Knockdown of MED26 resulted in little change in the levels of U7 snRNA. (c) Western blotting showing MED26 and Nucleolin expression 2h or 6h after Auxin treatment of MED26-AID expressing cell lines.

Supplementary Figure 2

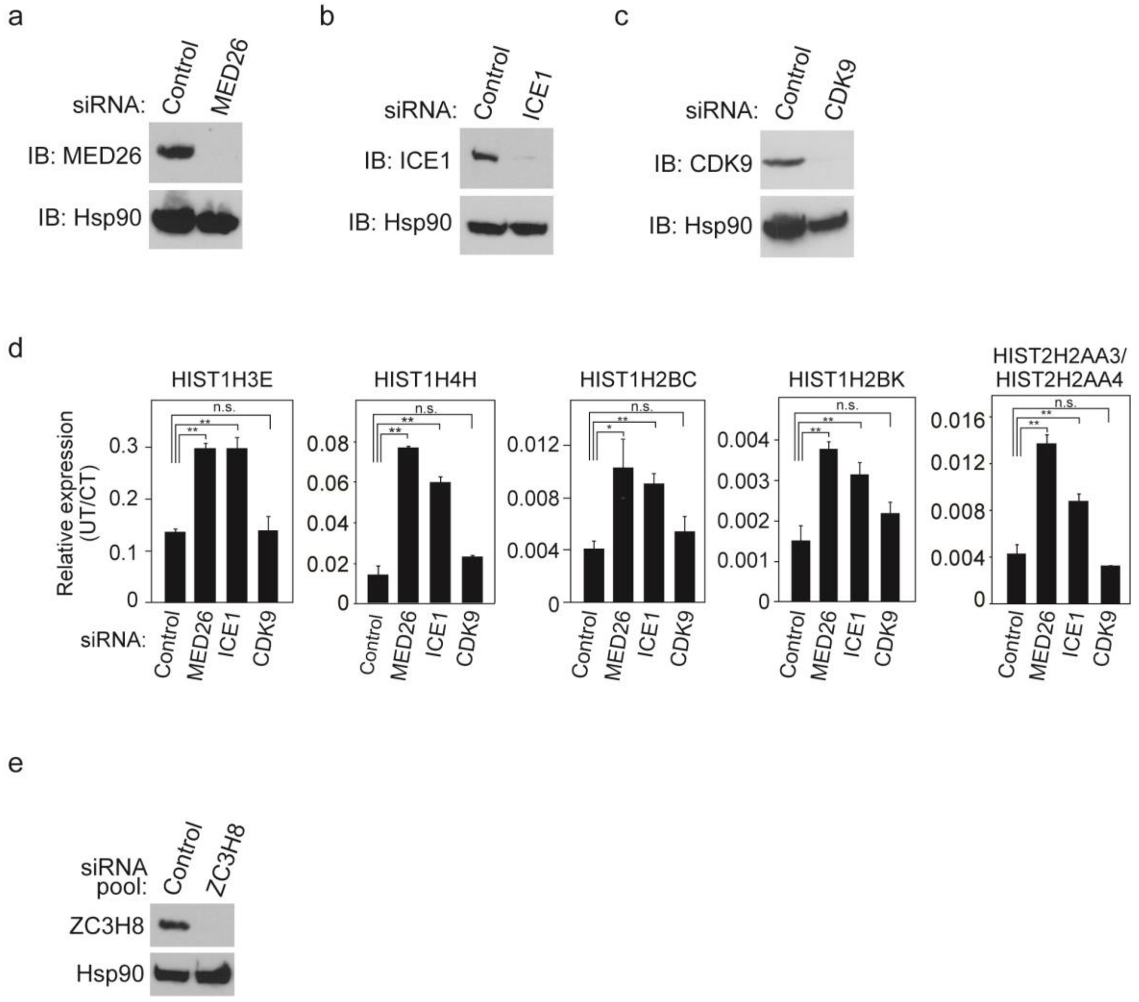

Supplementary Figure 2. LEC, but not SEC, is required for efficient 3'-processing of RDH genes. (a–c) Western blotting showing MED26, ICE1, CDK9, and Hsp90 expression 48 h after the transfection of nontargeting siRNA or siRNAs targeting each of MED26, ICE1, and CDK9. (d) Knockdown of MED26 and ICE1, but not CDK9, increased the ratio of unprocessed transcript (UT) and coding transcript (CT) of Replication-Dependent Histone (RDH) genes. Total RNAs were extracted from cells in which siRNA of each of control, MED26, ICE1, and CDK9 was transfected and the levels of the indicated transcripts were measured by real-time qPCR. Ct values were normalized to the coding transcript of the genes. Data points are the average of three independent experiments and error bars show standard deviation. The *P* values for the indicated comparisons were determined by Student's *t* test (*, $P < 0.05$; **, $P < 0.01$). n=3 biologically independent samples. (e) Western blotting showing ZC3H8 and Hsp90 expression 48 h after transfection of nontargeting siRNA or each of the siRNAs targeting ZC3H8.

Supplementary Figure 3

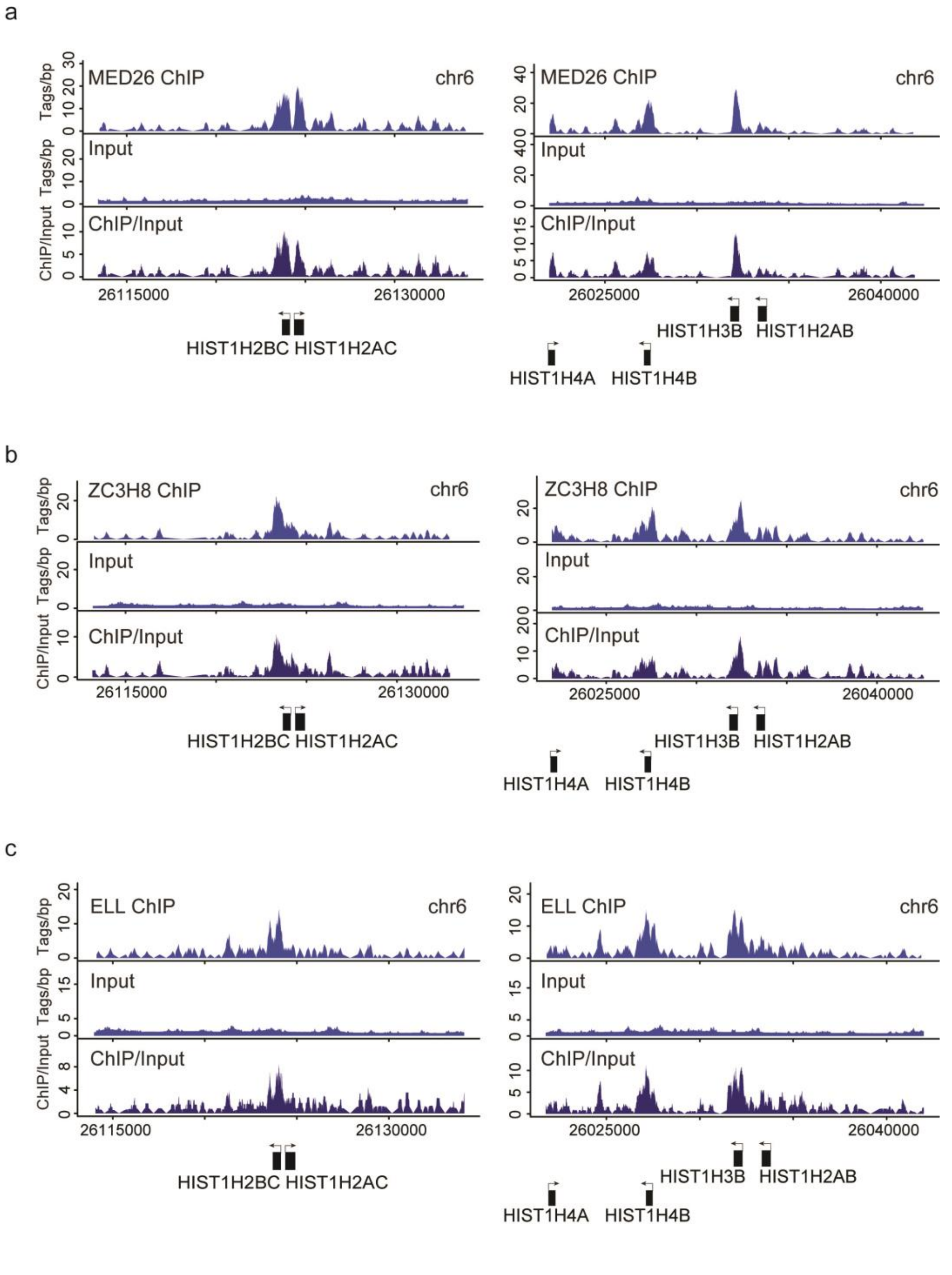

Supplementary Figure 3. MED26 and LEC are present at RDH genes. (a–c) Examples of ChIP-seq genome browser tracks showing the presence of MED26 (a), ZC3H8 (b), and ELL (c) at Replication-Dependent Histone (RDH) genes including *HIST1H2BC*, *HIST1H2AC*, *HIST1H3B*, *HIST1H2AB*, *HIST1H4A*, and *HIST1H4B*. ChIP-seq data of MED26 are from duplicate ChIP samples. ChIP-seq data of ELL and ZC3H8 are from encode data[1].

Supplementary Figure 4

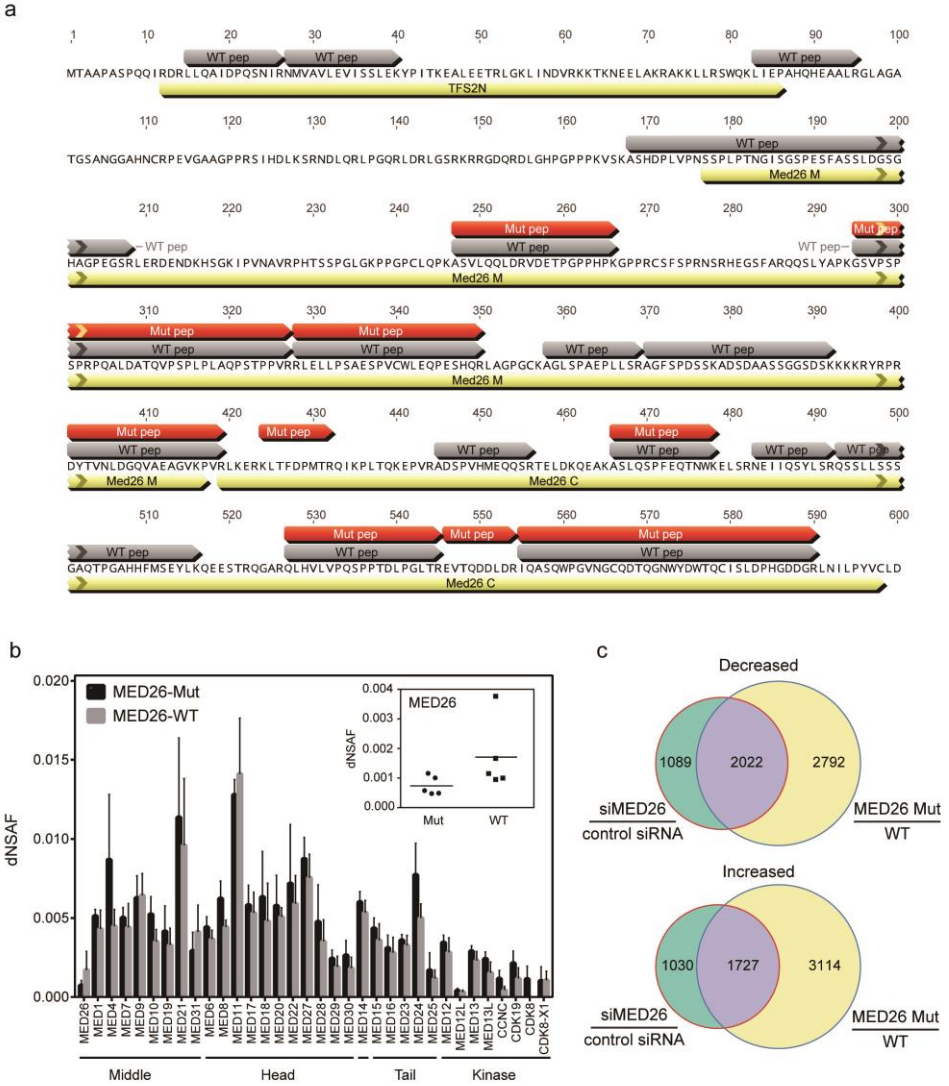

Supplementary Figure 4. Mediator assembly from the MED26 NTD mutant cell line. (a) MED26 sequence (NP_004822.2) showing peptides identified in Mediator-enriched fractions from HEK293 cells expressing wild type or mutant MED26. WT pep, peptides from wild type cells; Mut pep, peptides from MED26 mutant cells. The yellow bars show the positions of annotated MED26 regions. MED26 C, MED26 C-terminal region that assembles into Mediator; MED26 M, MED26 middle region; TFS2N, TFIIS-like MED26 NTD that interacts with LEC and SEC. (b) Comparison of Mediator from wild type and MED26 mutant cells. Mediator was enriched from lysates of wild type or MED26 mutant HEK293T cells on glutathione-Sepharose, using a GST a fusion protein containing the Mediator-binding activation domain of ATF6α (GST-ATF6α[1-150]) and analyzed by MudPIT mass spectrometry as described [2]. In a MudPIT dataset, the number of spectra from peptides of that protein is a function of its length and abundance. The relative abundance of a protein in different samples can be estimated from a normalized spectral abundance factor, or dNSAF [3]. The graph shows the mean and standard deviation of Mediator subunit dNSAFs from 5 independent affinity purifications. (c) Venn diagram showing overlap between genes that exhibit significant up- or down-regulation (FDR ≤ 0.05) after siRNA-mediated knockdown of MED26 or MED26 mutation, out of a total of 16,005 genes measured. The overlap in both cases is highly significant (hypergeometric tests of overlap, $p \approx 0$).

Supplementary Figure 5

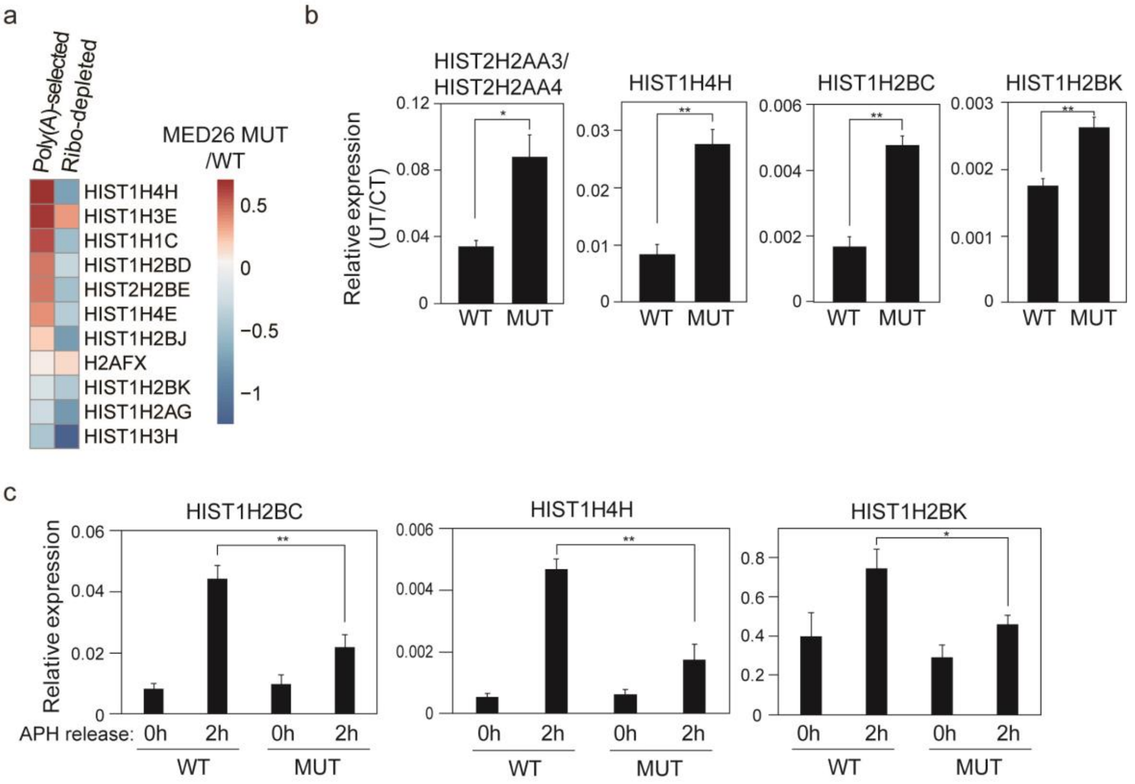

Supplementary Figure 5. The effect of MED26 NTD deletion on RDH genes transcription. (a) Heat map showing the differences in transcript abundance in wild-type (WT) and MED26 hypomorphic mutant (MUT) HEK293T cells ($\log_2$ MED26 MUT/WT), determined by RNA-seq of poly A-selected or ribo-depleted libraries. (b) The ratio of unprocessed transcripts (UT) and coding transcripts (CT) of RDH genes was increased in MED26 hypomorphic mutant (MUT) cells compared with wild-type cells. Data points are the mean of three independent experiments and error bars show the standard deviation. The *P* values for the indicated comparisons were determined by Student's *t* test (*, $P < 0.05$; **, $P < 0.01$). n=3 biologically independent samples. (c) Induction of RDH genes at S-phase was decreased in MED26 hypomorphic mutant cells. Wild-type (WT) and hypomorphic mutant (MUT) HEK293T cells were arrested at S-phase by aphidicolin (APH). After release from S-phase by removing APH, the cells were harvested at the indicated time. Total RNA was extracted from the cells and the ratio of RDH transcripts to GAPDH transcripts was measured by real-time qPCR. Data points are the mean of three independent experiments and error bars show the standard deviation. The *P* values for the indicated comparisons were determined by Student's *t* test (*, $P < 0.05$; **, $P < 0.01$). n=3 biologically independent samples.

Supplementary Figure 6

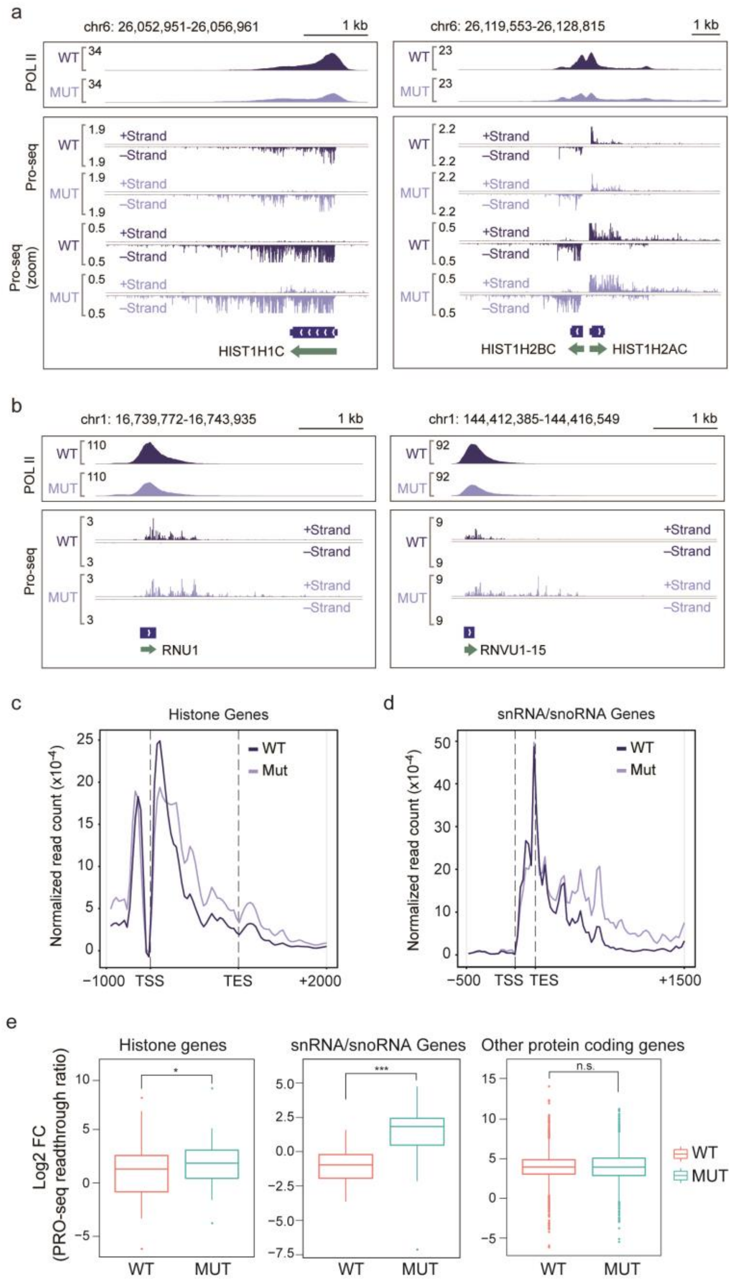

Supplementary Figure 6. Increased read-through transcripts of RDH genes and small nuclear RNA (snRNA) genes in cells expressing a MED26 hypomorph. (a, b) Precise run-on sequence analysis using HEK293T cells and mutant (MUT) cells. PRO-Seq genome browser tracks showing examples of Replication-Dependent Histone (RDH) genes (a) and snRNA genes (b) from wild-type or MED26 hypomorphic mutant cells. (c) Average gene plots of PRO-seq reads at RDH genes, showing increased PRO-seq reads downstream of TES in MED26 hypomorphic mutant (MUT) cells. (d) Average gene plots of PRO-seq reads at LEC-occupied snRNA and snoRNA genes, showing increased read-through transcripts in MED26 mutant (MUT) cells. (e) Calculation of PRO-seq readthrough ratios of transcripts for RDH, snRNA, snoRNA, and other protein-coding genes in HEK293T cells and MED26 hypomorphic mutant (MUT) cells. The P values for the indicated comparisons were determined by Student's t test (*, $P < 0.07$; **, $P < 0.01$; ***, $P < 0.001$). n.s.; not significant. Center line of each box plot represents the median. Upper fence and lower fence of each box plot represents upper and lower quartiles, respectively. The range of each whisker represents 1.5 times the interquartile range.

Supplementary Figure 7

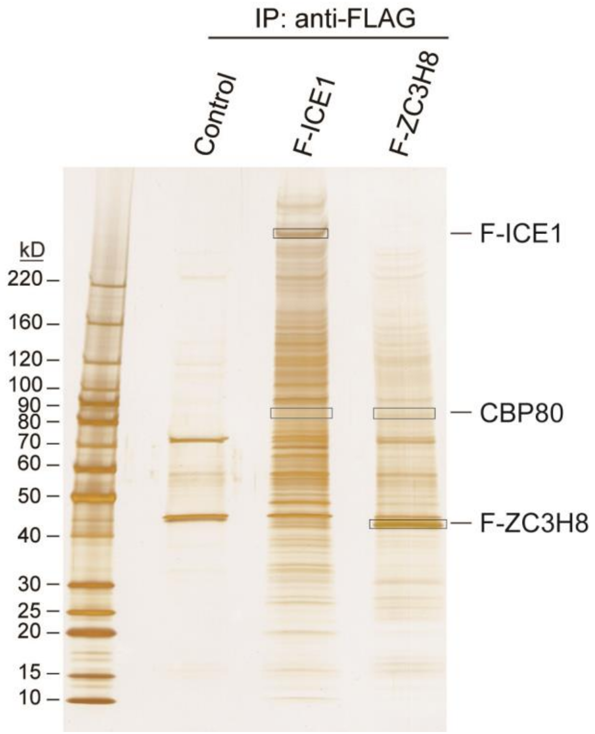

Supplementary Figure 7. Silver staining of F-ICE1 and F-ZC3H8 binding proteins purified by anti-FLAG affinity chromatography. The bands enclosed in the square were cut out from the gels and subjected to mass spectrometry analysis. The proteins identified by mass spectrometry are indicated.

Supplementary Figure 8

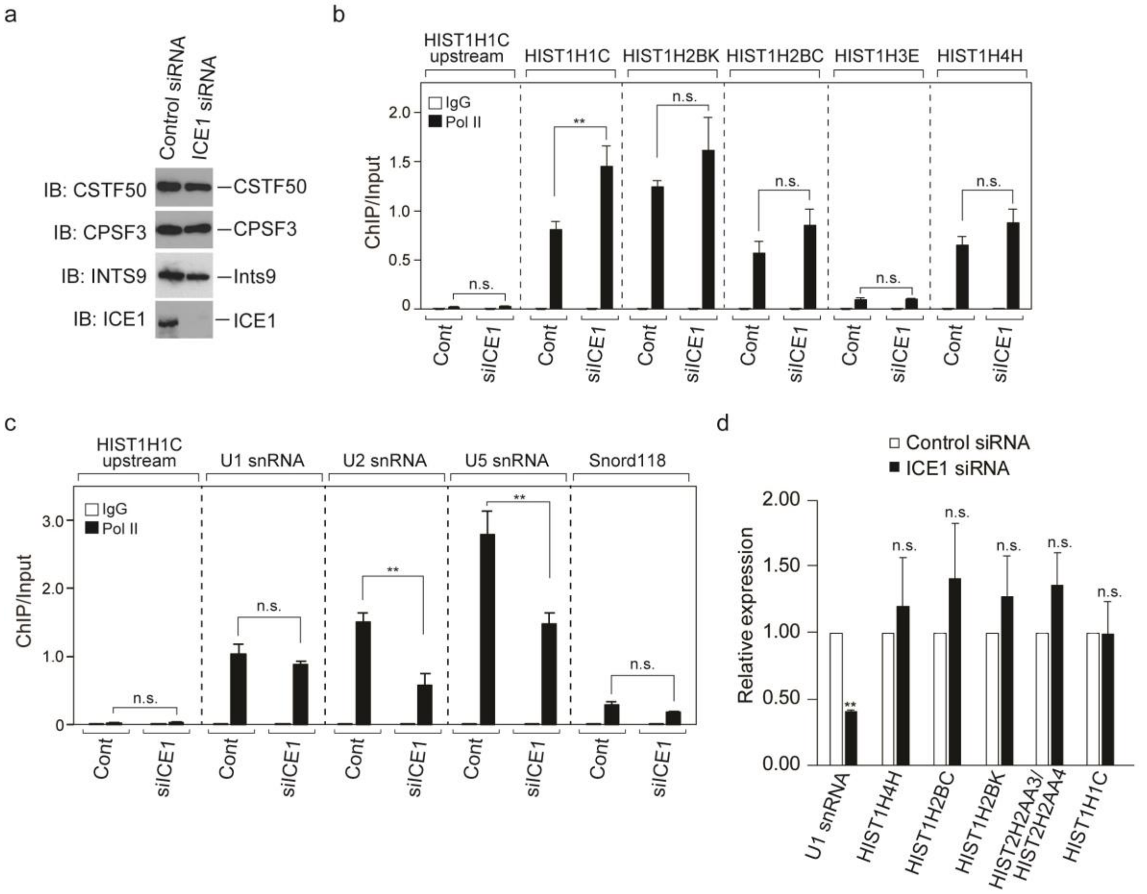

Supplementary Figure 8. Effect of ICE1 knockdown on occupancy of Pol II at RDH and snRNA genes. (a) Knockdown of ICE1 did not affect the expression of CSTF50, CPSF73, and INTS9. Western blotting analysis showed CSTF50, CPSF73, INTS9, and ICE1 expression at 48 h after transfection of non-targeting siRNA or siRNAs targeting ICE1. (b) Knockdown of ICE1 did not affect the occupancy of Pol II at RDH genes. The Ct values of each ChIP were normalized by that of the input. Each value is the mean of three independent experiments and error bars show the standard deviation. The *P* values for the indicated comparisons were determined by Student's *t* test (*, $P < 0.05$; **, $P < 0.01$). n=3 biologically independent samples. (c) Knockdown of ICE1 moderately decreased the occupancy of Pol II at snRNA genes. The Ct values of each ChIP were normalized by that of the input. Each value is the mean of three independent experiments and error bars show the standard deviation. The *P* values for the indicated comparisons were determined by Student's *t* test (*, $P < 0.05$; **, $P < 0.01$). n=3 biologically independent samples. (d) Knockdown of ICE1 did not affect the total transcripts of RDH genes, but moderately decreased the total transcripts of the U1 snRNA gene. Data points are the mean of three independent experiments and error bars show the standard deviation. The *P* values for the indicated comparisons were determined by Student's t test (*, $P < 0.05$; **, $P < 0.01$). n=3 biologically independent samples.

Supplementary Figure 9

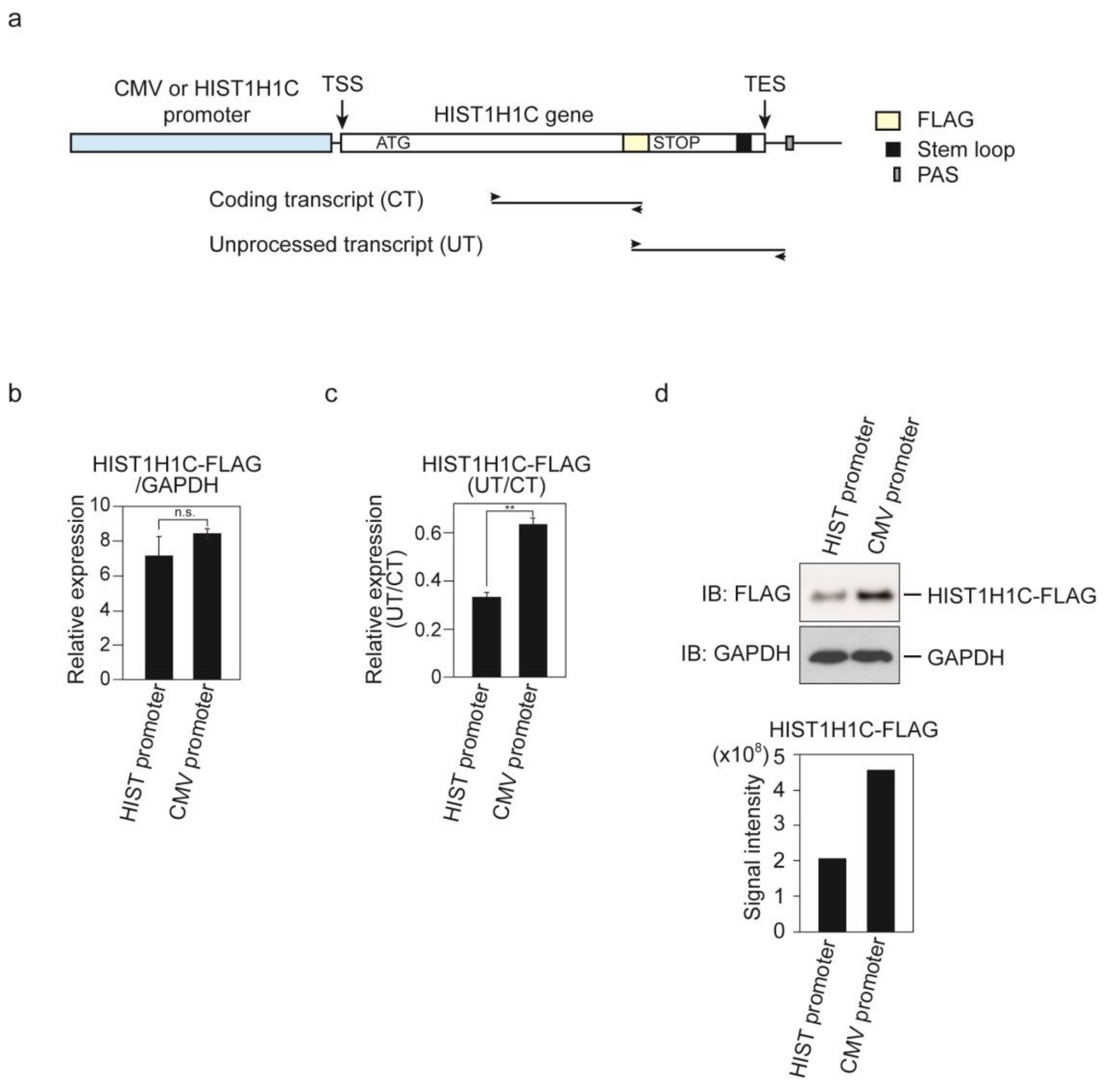

Supplementary Figure 9. Effect of replacing the RDH promoter with the CMV promoter on transcription termination of RDH genes. (a) Organization of the HIST1H1C gene with the HIST1H1C promoter or CMV promoter and amplified regions of coding transcripts (CT) and unprocessed transcripts (UT) in quantitative real-time PCR analysis. The 3'-end processing sites of RDH transcripts are localized between their stem-loop regions and downstream polyadenylation sites (PAS). A FLAG-tag sequence was added to the end of the HIST1H1C-coding region. (b) HEK293T cells were transiently transfected with each of the two plasmids encoding the HIST1H1C gene with the HIST1H1C promoter or CMV promoter. Total RNA was extracted from cells and the ratio of HIST1H1C-FLAG transcripts to GAPDH transcripts was measured by real-time qPCR. (c) The ratio of unprocessed transcripts (UT) to total transcripts (coding transcripts, CT) from each of the HIST1H1C-FLAG plasmids was measured by real-time qPCR. Data points are the mean of three independent experiments and error bars show the standard deviation. The P values for the indicated comparisons were determined by Student's t test (*, $P < 0.05$; **, $P < 0.01$). n=3 biologically independent samples. (d) Western blotting analysis showing HIST1H1C-FLAG and GAPDH expression at 48 h after transfection of each of the HIST1H1C-FLAG plasmids (upper panel). The signal intensity of the HIST1H1C-FLAG based was measured by ImageJ software (lower panel).

Supplementary Methods

Oligonucleotides information of primers used for qPCR analysis and CRISPR vector construction

Primers used for qPCR analysis

Human HIST2H2AA3/HIST2H2AA4 (CT)

5′-GCTCGGGACAACAAGAAGA-3′ (forward)

5′-CAGTACGGCCTGGATGTTAG-3′ (reverse)

Human HIST2H2AA3/HIST2H2AA4 (UT)

5′-TCCCTAAGAAGACGGAGAGT-3′ (forward)

5′-CAGCATTAACAACTCTTTTATTTG-3′ (reverse)

Human HIST1H4H (CT)

5′-CAAGCGAATTTCTGGCCTTATC-3′ (forward)

5′-TTTGGCGTGCTCTGTGTAA-3′ (reverse)

Human HIST1H4H (UT)

5′-GACAGCAATGGATGTGGTCTA-3′ (forward)

5′-TAACAAGATGTCTGCTCTTTTATG-3′ (reverse)

Human HIST1H2BK (CT)

5′-CTCTAAGGCCATGGGAATCAT-3′ (forward)

5′-GTCGAGCGCTTGTTGTAATG-3′ (reverse)

Human HIST1H2BK (UT)

5′-TGTCCGAGGGCACCAAG-3′ (forward)

5′-CACGTGTTTACAGCTCTAATATCG-3′ (reverse)

Human HIST1H2BC (CT)

5′-GACACTGGCATCTCTTCCAA-3′ (forward)

5′-GTCGAGCGCTTGTTGTAATG-3′ (reverse)

Human HIST1H2BC (UT)

5′-CACCAGCTCCAAGTAAACATTC-3′ (forward)

5′-CACAGCTCTTTTAGTGGGT-3′ (reverse)

Human HIST1H3E (CT)

5′-GCGAGAAATAGCTCAGGACTT-3′ (forward)

5′-CACGCGTTTGGCATGAATAG-3′ (reverse)

Human HIST1H3E (UT)

5′-GTGCGCTATTCATGCCAAAC-3′ (forward)

5′-ATTACAGCCCTTTTCACGGAC-3′ (reverse)

Human HIST1H1C upstream

5′-ATTCAGAGCTCTTAGGCGAAAT-3′ (forward)

5′-CATTGACACATACACAAGCTCAC-3′ (reverse)

Human HIST1H1C promoter

5′-AATGAAGAGCATGAAGCCCGAGG-3′ (forward)

5′-AACTCGGGTACAAGTGGCAAAGC-3′ (reverse)

Human HIST2H2AA4 promoter

5′-GGTGTTGTGTGCTCTCCTAAA-3′ (forward)

5′-GGCCATTTAACCACCTCAGT-3′ (reverse)

Human U1 snRNA

5′-ATACCATGATCACGAAGGTGGTT-3′ (forward)

5′-CAGTCCCCCACTACCACAAATTA-3′ (reverse)

Human U2 snRNA

5′-CTTCTCGGCCTTTTGGCTAAGAT-3′ (forward)

5′-GTACTGCAATACCAGGTCGATGC-3′ (reverse)

Human U5 snRNA

5′-CTCTGGTTTCTCTTCAGATCGCA-3′ (forward)

5′-TTGGGTTAAGACTCAGAGTTGTTCC-3′ (reverse)

Human SNORD118

5′-GAGGGCAGATTAGAACATGATGA-3′ (forward)

5′-GCAATCAGGGTGTTGCAAGT-3′ (reverse)

Human GAPDH

5′-TCGACAGTCAGCCGCATCTTCTTT-3′ (forward)

5′-GCCCAATACGACCAAATCCGTTGA-3′ (reverse)

Primers used for plasmid based HIST1H1C qPCR analysis

FLAG-HIST1H1C (UT)

5′-ATGCCAGACTACAAGGACGATGAT-3′ (forward)

5′-AAGTAACAGGGCAGAACAAGAAAAG-3′ (reverse)

FLAG-HIST1H1C (CT)

5′-GCCAAAAGTGCTGCTAAGGCTGT-3′ (forward)

5′-ATCATCGTCCTTGTAGTCTGGCAT-3′ (reverse)

Oligonucleotides used for CRISPR vector construction

MED26 sgRNA2-Sense

CACCGCGGTCGCACGGCGTTGACG

MED26 sgRNA2-Antisense

AAACCGTCAACGCCGTGCGACCGC

MED26 sgRNA3-Sense

CACCGCTTGGCGAGCTCCTCGTTCT

MED26 sgRNA3-Antisense

AAACAGAACGAGGAGCTCGCCAAGC

Supplementary References

1. Hu D*, et al.* The little elongation complex functions at initiation and elongation phases of snRNA gene transcription. *Mol Cell* **51**, 493-505 (2013).

2. Sela D*, et al.* Endoplasmic reticulum stress-responsive transcription factor ATF6alpha directs recruitment of the Mediator of RNA polymerase II transcription and multiple histone acetyltransferase complexes. *The Journal of biological chemistry* **287**, 23035-23045 (2012).

3. Zybailov B, Mosley AL, Sardiu ME, Coleman MK, Florens L, Washburn MP. Statistical analysis of membrane proteome expression changes in Saccharomyces cerevisiae. *Journal of proteome research* **5**, 2339-2347 (2006).
