# Supplementary material for: The role of Mediator and Little Elongation Complex in transcription termination
Source: Nat Commun. 2020 Feb 26;11:1063. doi: 10.1038/s41467-020-14849-1 (PMC7044329; doi:10.1038/s41467-020-14849-1)
Supplement: Supplementary file 3 — Description of Additional Supplementary Files [file 41467_2020_14849_MOESM3_ESM.pdf]

Description of Additional Supplementary Files

File Name: Supplementary Data 1

Description: Supplementary Data 1 is related to Figure 1. RNA-seq of PolyA-selected and ribodepleted libraries from HEK293T cells transfected with either control siRNA or MED26 siRNA or HEK293T cells expressing wild type MED26 or MED26 mutants lacking the NTD. The data includes transcripts exhibiting increased abundance (log2 ≥ 1.5, FDR ≤ 0.05) in polyA-selected libraries.

File Name: Supplementary Data 2

Description: Supplementary Data 2 is related to Figure 2. Oligo-dT selection-based RNA-seq analysis using HEK293T cells transfected with either control siRNA, MED26 siRNA, ICE1 siRNA or AFF4 siRNA.

File Name: Supplementary Data 3

Description: Supplementary Data 3 is related to Figure 3. Gene annotation of MED26, ZC3H8 and ELL ChIP-sequence analysis.

File Name: Supplementary Data 4

Description: Supplementary Data 4 is related to Figure 4. RNA-seq of polyA-selected libraries from HEK293T cells transfected with control siRNA or MED26 siRNA. The data includes snRNA/snoRNA transcripts exhibiting significance (FDR ≤ 0.1) in polyA-selected libraries.
