# Supplementary material for: The role of Mediator and Little Elongation Complex in transcription termination
Source: Nat Commun. 2020 Feb 26;11:1063. doi: 10.1038/s41467-020-14849-1 (PMC7044329; doi:10.1038/s41467-020-14849-1)
Supplement: Supplementary file 8 — Reporting Summary [file 41467_2020_14849_MOESM8_ESM.pdf]

nature research

Corresponding author(s): Hidehisa Takahashi
Joan W. Conaway
Shigetsugu Hatakeyama

Last updated by author(s): Jan 21, 2020

Reporting Summary

Nature Research wishes to improve the reproducibility of the work that we publish. This form provides structure for consistency and transparency in reporting. For further information on Nature Research policies, see Authors & Referees and the Editorial Policy Checklist.

Statistics

For all statistical analyses, confirm that the following items are present in the figure legend, table legend, main text, or Methods section.

n/a	Confirmed	
☐	☒	The exact sample size (*n*) for each experimental group/condition, given as a discrete number and unit of measurement
☐	☒	A statement on whether measurements were taken from distinct samples or whether the same sample was measured repeatedly
☐	☒	The statistical test(s) used AND whether they are one- or two-sided<br>*Only common tests should be described solely by name; describe more complex techniques in the Methods section.*
☐	☒	A description of all covariates tested
☐	☒	A description of any assumptions or corrections, such as tests of normality and adjustment for multiple comparisons
☐	☒	A full description of the statistical parameters including central tendency (e.g. means) or other basic estimates (e.g. regression coefficient) AND variation (e.g. standard deviation) or associated estimates of uncertainty (e.g. confidence intervals)
☐	☒	For null hypothesis testing, the test statistic (e.g. *F*, *t*, *r*) with confidence intervals, effect sizes, degrees of freedom and *P* value noted<br>*Give P values as exact values whenever suitable.*
☒	☐	For Bayesian analysis, information on the choice of priors and Markov chain Monte Carlo settings
☒	☐	For hierarchical and complex designs, identification of the appropriate level for tests and full reporting of outcomes
☒	☐	Estimates of effect sizes (e.g. Cohen's *d*, Pearson's *r*), indicating how they were calculated

Our web collection on statistics for biologists contains articles on many of the points above.

Software and code

Policy information about availability of computer code

Data collection

All datas were collected by the experiments indicated in the manuscript.

Data analysis

For ChIP-seq analysis, sequencing reads were acquired through primary Solexa image analysis. Filtered reads were then aligned either to the human genome (hg38) for MED26 ChIP or to an mm10-hg38 combined genome for Rpb1 ChIP using the Bowtie alignment tool. Only those sequences that matched uniquely to the genome with up to two mismatches and mapped to fewer than three locations were retained for subsequent analyses. The public data were downloaded from GEO database with accession ID GSE47938. Reads were aligned to human genome hg38 using Bowtie2 (version 2.2.4) with default settings. Peaks were called using MACS2 callpeak (version 2.1.1), default parameter, based on the corresponding input samples.

For RNA-seq analysis, raw reads were demultiplexed allowing up to one mismatch using Illumina bcl2fastq2 v2.18. Reads were then mapped to human genome hg38, with STAR aligner (version 2.5.3a) default settings, using Ensembl 87 annotation gene models. Transcripts per kilobase millon (TPM) values were then generated using RSEM (version 1.3.0) function rsem-calculate-expression with option –estimate-rspd. Differential gene expression analysis was performed using R (v3.22.3) package edgeR (v3.5.0).

For PRO-seq analysis, raw reads were demultiplexed allowing up to one mismatch using Illumina bcl2fastq2 v2.18. The adaptor sequence was removed, and reads were trimmed to 36 bp. After reverse-complementing, reads were aligned to the dm6-hg38 combined genome using Bowtie2 (version 2.2.4) with default settings.

For manuscripts utilizing custom algorithms or software that are central to the research but not yet described in published literature, software must be made available to editors/reviewers. We strongly encourage code deposition in a community repository (e.g. GitHub). See the Nature Research guidelines for submitting code & software for further information.

Data

Policy information about availability of data

All manuscripts must include a data availability statement. This statement should provide the following information, where applicable:

- Accession codes, unique identifiers, or web links for publicly available datasets
- A list of figures that have associated raw data
- A description of any restrictions on data availability

ChIP-seq, RNA-seq and PRO-seq data for are deposited in GEO under accession GSE121024.
(https://www.ncbi.nlm.nih.gov/geo/query/acc.cgi?acc=GSE121024).

A list of Figures that have associated raw data.
293T-D2G8-MED26-MUT_Rpb1_Input_Rep1.fastq.gz: Figure 4a-h, Figure S5b-d
293T-D2G8-MED26-MUT_Rpb1_Input_Rep2.fastq.gz: Figure 4a-h, Figure S5b-d
293T-D2G8-MED26-MUT_Rpb1-IP_Rep1.fastq.gz: Figure 4a-h, Figure S5b-d
293T-D2G8-MED26-MUT_Rpb1-IP_Rep2.fastq.gz: Figure 4a-h, Figure S5b-d
293T-WT_Rpb1_Input_Rep1.fastq.gz: Figure 4a-h, Figure S5b-d
293T-WT_Rpb1_Input_Rep2.fastq.gz: Figure 4a-h, Figure S5b-d
293T-WT_Rpb1_IP_Rep1.fastq.gz: Figure 4a-h, Figure S5b-d
293T-WT_Rpb1_IP_Rep2.fastq.gz: Figure 4a-h, Figure S5b-d
293T_Input_MED26_ChIP_Rep1.fastq.gz: Figure 3a-c, Figure S3a, Table S3
293T_Input_MED26_ChIP_Rep2.fastq.gz: Figure 3a-c, Figure S3a, Table S3
293T_Med26_ChIP_Rep1.fastq.gz: Figure 3a-c, Figure S3a, Table S3
293T_Med26_ChIP_Rep2.fastq.gz: Figure 3a-c, Figure S3a, Table S3
Proseq_293T_D2G8-MED26-MUT_Rep1.fastq.gz: Figure 4a-h, Figure S5b-d
Proseq_293T_D2G8-MED26-MUT_Rep2.fastq.gz: Figure 4a-h, Figure S5b-d
Proseq_293T_WT_Rep1.fastq.gz: Figure 4a-h, Figure S5b-d
Proseq_293T_WT_Rep2.fastq.gz: Figure 4a-h, Figure S5b-d
293T_AFF4_KD_polyA_Rep1.fastq.gz: Figure 2c,d, Table S2
293T_AFF4_KD_polyA_Rep2.fastq.gz: Figure 2c,d, Table S2
293T_AFF4_KD_polyA_Rep3.fastq.gz: Figure 2c,d, Table S2
293T_Control_polyA_Rep1.fastq.gz: Figure 1a, Figure 2c,d, Figure S5a, Table S1, Table S2
293T_Control_polyA_Rep2.fastq.gz: Figure 1a, Figure 2c,d, Figure S5a, Table S1, Table S2
293T_Control_polyA_Rep3.fastq.gz: Figure 1a, Figure 2c,d, Figure S5a, Table S1, Table S2
293T_KIAA0947-ICE1_KD_polyA_Rep1.fastq.gz: Figure 2c,d, Table S2
293T_KIAA0947-ICE1_KD_polyA_Rep2.fastq.gz: Figure 2c,d, Table S2
293T_KIAA0947-ICE1_KD_polyA_Rep3.fastq.gz: Figure 2c,d, Table S2
293T-D2G8-MED26-MUT_Ribodep_Rep1.fastq.gz: Figure S5a, Table S1
293T-D2G8-MED26-MUT_Ribodep_Rep2.fastq.gz: Figure S5a, Table S1
293T-D2G8-MED26-MUT_Ribodep_Rep3.fastq.gz: Figure S5a, Table S1
293T-D2G8-MED26-MUT_Ribodep_Rep4.fastq.gz: Figure S5a, Table S1
293T_MED26_KD_polyA_Rep1.fastq.gz: Figure 1a, Figure S5a, Table S1
293T_MED26_KD_polyA_Rep2.fastq.gz: Figure 1a, Figure S5a, Table S1
293T_MED26_KD_polyA_Rep3.fastq.gz: Figure 1a, Figure S5a, Table S1
293T_MED26_KD_Ribodep_Rep1.fastq.gz: Figure 1a, Figure S5a, Table S1
293T_MED26_KD_Ribodep_Rep2.fastq.gz: Figure 1a, Figure S5a, Table S1
293T_MED26_KD_Ribodep_Rep3.fastq.gz: Figure 1a, Figure S5a, Table S1
293T_Control_Ribodep_Rep1.fastq.gz: Figure 1a, Figure S5a, Table S1
293T_Control_Ribodep_Rep2.fastq.gz: Figure 1a, Figure S5a, Table S1
293T_Control_Ribodep_Rep3.fastq.gz: Figure 1a, Figure S5a, Table S1
293T_WT_Ribodep_Rep1.fastq.gz: Figure 1a, Figure S5a, Table S1
293T_WT_Ribodep_Rep2.fastq.gz: Figure 1a, Figure S5a, Table S1
293T_WT_Ribodep_Rep3.fastq.gz: Figure 1a, Figure S5a, Table S1
293T_WT_Ribodep_Rep4.fastq.gz: Figure 1a, Figure S5a, Table S1

The mass spectrometric datasets have been deposited to the ProteomeXchange via the MassIVE repository.
(ftp://massive.ucsd.edu/MSV000083465).

Original data underlying parts of this study performed at the Stowers Institute can be downloaded from the Stowers Original Data Repository.
(http://www.stowers.org/research/publications/LIBPB-1361)

Field-specific reporting

Please select the one below that is the best fit for your research. If you are not sure, read the appropriate sections before making your selection.

☒ Life sciences ☐ Behavioural & social sciences ☐ Ecological, evolutionary & environmental sciences

For a reference copy of the document with all sections, see nature.com/documents/nr-reporting-summary-flat.pdf

Life sciences study design

All studies must disclose on these points even when the disclosure is negative.

Sample size	Triplicate samples were used for all of the experiments except ChIp-seq analysis. Duplicate samples were used for ChIP-seq analysis.
Data exclusions	There is no data excluded from the analysis.
Replication	We performed at least 3 times experiments to verify the reproducibility of each experiment. We confirmed that all attempts were successful.
Randomization	Randomization is not relevant to our research, since this research is not relevant to clinical trial study.
Blinding	Blinding is not relevant to our research, since this research is not relevant to clinical study.

Reporting for specific materials, systems and methods

We require information from authors about some types of materials, experimental systems and methods used in many studies. Here, indicate whether each material, system or method listed is relevant to your study. If you are not sure if a list item applies to your research, read the appropriate section before selecting a response.

Materials & experimental systems

n/a	Involved in the study	
☐	☒	Antibodies
☐	☒	Eukaryotic cell lines
☒	☐	Palaeontology
☒	☐	Animals and other organisms
☒	☐	Human research participants
☒	☐	Clinical data

Methods

n/a	Involved in the study	
☐	☒	ChIP-seq
☒	☐	Flow cytometry
☒	☐	MRI-based neuroimaging

Antibodies

Antibodies used

Western blotting.
Anti-FLAG M2 antibodies (1:2000 dilution; Sigma-Aldrich Corp.), anti-HA antibodies (1:2000 dilution; Covance), anti-MED26 antibodies (1:1000 dilution, sc-48776 X; Santa Cruz Biotechnology), anti-ICE1 antibodies (1:200 dilution, HPA054452; Sigma-Aldrich Corp.), anti-CBP80 antibodies (1:1000 dilution, 24964S; Cell Signaling Technology), anti-CBP20 antibodies (1:200 dilution, sc-48793; Santa Cruz), anti-ZC3H18 antibodies (1:1000 dilution, A304-682A; Bethyl Laboratories), anti-ARS2 antibodies (1:1000 dilution, ab192999; Abcam), anti-SLBP antibodies (1:1000 dilution, RN045P; Medical & Biological Laboratories Co., Ltd.), anti-NELFb antibodies (1:2000 dilution, sc-5334 X; Santa Cruz), anti-NELFe antibodies (1:200 dilution, ab170104; Abcam), anti-Symplekin antibodies (1:1000 dilution, ab80274; Abcam), anti-CPSF1 antibodies (1:200 dilution, ab81552; Abcam), anti-CSTF50 antibodies (1:1000 dilution, A301-250A; Bethyl Laboratories), anti-CSTF64 antibodies (1:1000 dilution, A301-092A; Bethyl Laboratories), anti-CPSF73 antibodies (1:200 dilution, A301-091A; Bethyl Laboratories), anti-INTS4 antibodies (1:1000 dilution, ab75253; Abcam), anti-INTS9 antibodies (1:1000 dilution, 13945S; Cell Signaling Technology), anti-ZC3H8 antibodies (1:1000 dilution, ab97821; Abcam), anti-CDK9 antibodies (1:200 dilution, sc-13130; Santa Cruz), anti-nucleolin antibodies (1:1000 dilution, ab13541; Abcam) and anti-AFF4 antibodies (1:1000 dilution, A302-538A; Bethyl Laboratories) were used.

ChIP assays.
The specific antibodies used were as follows: MED26 (H-228, sc-48776 X; Santa Cruz), ICE1 (A304-276A; Bethyl Laboratories), ELL (A301-645A; Bethyl Laboratories), CPSF73 (A301-091A; Bethyl Laboratories), INTS9 (anti-RC74, A300-412A; Bethyl Laboratories), and Rpb1 (D8L4Y, 14958S; Cell Signaling Technology).

Immunostaining.
Primary antibodies to coilin (1:2000 dilution, ab11822, abcam), MED26 (1:500 dilution, D4B1, 14950S, Cell Signaling Technology), MED6 (1:100 dilution, sc-9433, Santa Cruz Biotechnology), MED24 (1:100 dilution, C-16, sc5338, Santa Cruz Biotechnology), NELFb (1:100 dilution, D6K9A, #14894, Cell signaling Technology), CSTF64 (1:100 dilution, A301-092A, Bethyl Laboratories), AFF4 (1:100 dilution, A302-539A, Bethyl Laboratories) and ICE1 (KIAA0947) (1:200 dilution, A304-276A, Bethyl Laboratories) were used in PBST containing 0.1% BSA.

Validation

Validation of each primary antibody are stated in manufacturer's website. We used each antibody following to the application stated in manufacturer's website.

Eukaryotic cell lines

Policy information about cell lines

Cell line source(s)	Human embryonic kidney 293T cell lines (HEK293T), Flp-In 293 cell lines, HeLa cell lines, HCT116 cell lines
Authentication	All of the cell lines used in experiments are not found in the cell lines registered as misidentified cell lines in the International Cell Line Authentication Committee (ICLAC).
Mycoplasma contamination	We confirmed that all of the cell lines were negative for mycoplasma contamination.
Commonly misidentified lines (See ICLAC register)	There is no commonly misidentified cell lines in this research.

ChIP-seq

Data deposition

☒ Confirm that both raw and final processed data have been deposited in a public database such as GEO.

☒ Confirm that you have deposited or provided access to graph files (e.g. BED files) for the called peaks.

Data access links<br>*May remain private before publication.*	ChIP-seq, RNA-seq and PRO-seq data for are deposited in GEO under accession GSE121024.<br>(https://www.ncbi.nlm.nih.gov/geo/query/acc.cgi?acc=GSE121024).
Files in database submission	293T-D2G8-MED26-MUT_Rpb1_Input_Rep1.fastq.gz<br>293T-D2G8-MED26-MUT_Rpb1_Input_Rep2.fastq.gz<br>293T-D2G8-MED26-MUT_Rpb1-IP_Rep1.fastq.gz<br>293T-D2G8-MED26-MUT_Rpb1-IP_Rep2.fastq.gz<br>293T-WT_Rpb1_Input_Rep1.fastq.gz<br>293T-WT_Rpb1_Input_Rep2.fastq.gz<br>293T-WT_Rpb1_IP_Rep1.fastq.gz<br>293T-WT_Rpb1_IP_Rep2.fastq.gz<br>293T_Input_MED26_ChIP_Rep1.fastq.gz<br>293T_Input_MED26_ChIP_Rep2.fastq.gz<br>293T_Med26_ChIP_Rep1.fastq.gz<br>293T_Med26_ChIP_Rep2.fastq.gz
Genome browser session<br>(e.g. UCSC)	http://genome.ucsc.edu/s/shc/GSE121024

Methodology

Replicates	In each experiment, two replicates samples are subjected to ChIP-seq analysis.
Sequencing depth	ChIP-seq of Rpb1<br>293T-D2G8-M26_Input_Rep1: Total number of reads 80838688, uniquely mapped reads 53911223, 75 single-end<br>293T-D2G8-M26_Input_Rep2: Total number of reads 66399306, uniquely mapped reads 46210009, 75 single-end<br>293T-D2G8-M26_IP_Rep1:Total number of reads 59878912, uniquely mapped reads 41650000, 75 single-end<br>293T-D2G8-M26_IP_Rep2:Total number of reads 61577954, uniquely mapped reads 44766389, 75 single-end<br>293T-WT-M26_Input_Rep1: Total number of reads 64375789, uniquely mapped reads 40899326, 75 single-end<br>293T-WT-M26_Input_Rep2: Total number of reads 63245460, uniquely mapped reads 41705577, 75 single-end<br>293T-WT-M26_IP_Rep1: Total number of reads 52019368, uniquely mapped reads 35202922, 75 single-end<br>293T-WT-M26_IP_Rep2: Total number of reads 62753864, uniquely mapped reads 43976538, 75 single-end<br><br>ChIP-seq of MED26<br>293T_Input_MED26_ChIP_Rep1, Total number of reads 70347697, uniquely mapped reads 47699411, 36 single-end<br>293T_Input_MED26_ChIP_Rep2, Total number of reads 70519977, uniquely mapped reads 47027208, 36 single-end<br>293T_Med26_ChIP_Rep1, Total number of reads 27687998, uniquely mapped reads 16426392, 36 single-end<br>293T_Med26_ChIP_Rep2, Total number of reads 27784793, uniquely mapped reads 16610123, 36 single-end
Antibodies	MED26 (H-228, sc-48776 X; Santa Cruz)<br>Rpb1 (D8L4Y, 14958S; Cell Signaling Technology)
Peak calling parameters	Peaks were called using MACS2 callpeak (version 2.1.1), default parameter, based on the corresponding input samples.
Data quality	Peaks with q score < 1e-4 were used for downstream analysis.<br>All of the ChIP-seq samples have more than 75% unique mapped reads to the genome.
Software	Reads were aligned to human genome hg38 using Bowtie2 (version 2.2.4) with default settings. Peaks were called using MACS2 callpeak (version 2.1.1), default parameter, based on the corresponding input samples.
